# Outcomes of surgery for acute type A dissection in octogenarians versus non-octogenarians: a systematic review and meta analysis

**DOI:** 10.1186/s13019-022-01980-x

**Published:** 2022-09-01

**Authors:** Aditya Eranki, Michael Merakis, Michael L. Williams, Campbell D. Flynn, Claudia Villanueva, Ashley Wilson-Smith, Yangsin Lee, Ross Mejia

**Affiliations:** 1 Department of Cardiothoracic Surgery, John Hunter Hospital, Newcastle, Australia; 2 School of Medicine and Public Health, University of Newcastle, Newcastle, Australia; 3 Department of Cardiothoracic Surgery, St George Hospital, Sydney, Australia; 4 The Collaborative Research (CORE) Group, Macquarie University, Sydney, Australia

**Keywords:** Type A aortic dissection, Octogenarian, Outcomes, Mid-term survival

## Abstract

**Introduction:**

Acute Type A Aortic Dissection (ATAAD) is a cardiothoracic emergency that requires urgent intervention. Elderly status, particularly age over 80, is an independent risk factor for mortality and morbidity. The mid-term outcomes of this age group are also unknown. This systematic review and meta-analysis of observational studies was therefore performed to analyse short- and mid-term mortality and morbidity in octogenarians following surgery for ATAAD.

**Methods:**

A systematic review was conducted for studies published since January 2000. The primary endpoint was short-term mortality, either reported as 30-day mortality or in-hospital mortality and medium-term (five year) survival. Secondary endpoints were rates of postoperative complications, namely stroke, acute renal failure (ARF), re-exploration and intensive care unit (ICU) length of stay (LOS).

**Results:**

A total of 16 retrospective studies, with a total of 16, 641 patients were included in the systematic review and meta-analysis. Pooled analysis demonstrated that octogenarian cohorts are at significantly higher risk of short-term mortality than non-octogenarians (OR 1.93; 95% CI 1.33–2.81; *P* < 0.001). Actuarial survival was significantly lower in the octogenarian cohort, with a five-year survival in the octogenarian cohort of 54% compared to 76% in the non-octogenarian cohort (*P* < 0.001). There were no significant differences between the cohorts in terms of secondary outcomes: stroke, ARF, re-exploration or ICU LOS.

**Conclusion:**

Octogenarians are twice as likely to die in the short-term following surgery for ATAAD and demonstrate a significantly lower five-year actuarial survival. Patients and family members should be well informed of the risks of surgery and suitable octogenarians selected for surgery.

**Supplementary Information:**

The online version contains supplementary material available at 10.1186/s13019-022-01980-x.

## Introduction

Acute type A aortic dissection (ATAAD) is a cardiothoracic emergency and is associated with a high morbidity and mortality [1–3]. The short-term postoperative mortality rate is between 15–30% and postoperative complications such as stroke, renal failure, tamponade and limb ischemia occur in up to one in four patients [1, 2]. The mainstay of management is urgent cardiothoracic intervention. Non-operative management confers a high short-term mortality rate and is usually reserved for patients who either refuse surgery or where surgery does not offer a good long-term prognosis [3]. Octogenarian status has been shown to be an independent risk factor for post-operative mortality in ATAAD [4]. Furthermore, they are more likely to present late with malperfusion, as a result of complex tears [4]. Despite this, surgery is being offered to this demographic as a result of an ageing population coupled with enhancements of surgical technique and post-operative care [5, 6]. The short-term and mid-term outcomes in this cohort are still unclear. A systematic review published in 2011 demonstrated that octogenarians have a higher short-term mortality [7]. Another published in 2016, defining an elderly cohort as age greater than 70, demonstrated short-term mortality two times higher in this cohort [8]. Since then, several well-designed retrospective studies have been published comparing outcomes of octogenarians to non-octogenarians following surgery for ATAAD. Our aim was to summarise the evidence comparing octogenarians to non-octogenarians following surgery for ATAAD, investigating short-term mortality and morbidity, as well as mid-term survival (5 years).

## Methods

### Search strategy and study selection

This review was written in accordance with Preferred Reporting Items for Systematic Reviews and Meta-Analyses (PRISMA) recommendations and guidance [9]. An electronic literature search was performed utilising PubMed, Scopus and OVID/MEDLINE databases from January 2000 to October 2021. The search strategy included a combination of keywords and Medical Subject Headings (MeSH) including “Type A Aortic Dissection” AND “Octogenarian” AND “Outcomes” NOT “Type B Aortic Dissection”. A total of 545 abstracts were screened after duplicates (51) were removed. Strict inclusion criteria below were applied, and 75 articles were selected for full text review. Two reviewers (A.E, M.M) assessed the eligibility of the selected papers. Discrepancies between full text reviews were adjudicated by a third reviewer (A.W.S). References of included articles were also crosschecked to see if additional studies could be identified. A total of 16 studies were included in the systematic review and meta-analysis. The search strategy is presented in Additional file 1: Fig. 1.

**Fig. 1:**
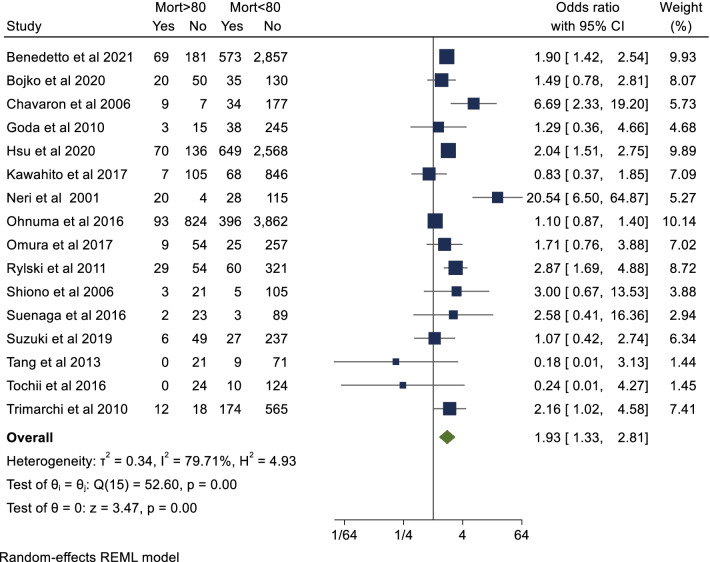
Short-term mortality

### Inclusion and exclusion criteria

The inclusion criteria were (1) Stanford Type-A Aortic Dissection, surgically managed (2) clearly defined octogenarian and non-octogenarian cohorts (3) short-term mortality and/or postoperative outcomes provided for both octogenarian and non-octogenarian cohorts (4) and published after year 2000. Studies that did not provide a comparison of mortality between octogenarian and non-octogenarian cohorts were excluded. All publications were limited to English. Conference abstracts, case reports, editorials, reviews and expert opinion pieces were excluded.

### Primary and secondary endpoints, study quality appraisal

The primary endpoint for this systematic review was short-term mortality, defined as either 30-day mortality or in-hospital mortality, and medium term (five year) survival. Secondary endpoints were rates of postoperative complications, namely stroke, acute renal failure (ARF), re-exploration and intensive care unit (ICU) length of stay (LOS) in days. ARF was uniformly defined as de-novo dialysis postoperatively (either temporarily or permanently). Study quality was assessed using the National Heart, Lung, and Blood Institute (NHLBI) Study Quality Assessment Tool [10]. Relevant aspects of each study were summarised on a table and study quality then assigned (Additional file 1: Table 1).

**Table 1:** Descriptive patient data

Variable		n (patients)	Mean ± SD	*P* value
Cross clamp time (min)	> 80YO	251	110 ± 43	*P* = 0.04
	< 80YO	1006	118 ± 58	
CPBT (min)	> 80YO	306	167 ± 51	*P* = 0.02
	< 80YO	1270	177 ± 68	
Aortic root replacement	> 80YO	52/1597 (3.3%)	–	*P* < 0.01
	< 80YO	734/9948 (7.4%)	–	
Total arch replacement	> 80YO	381/1597 (24%)	–	*P* = 0.01
	< 80YO	3256/9948 (33%)	–	
DHCA	> 80YO	202	30.5 ± 12.9	*P* = 0.37
	< 80YO	781	31.2 ± 15.5	
ACP	> 80YO	79/186 (42%)	–	*P* = 0.04
		428/727 (59%)	–	
Malperfusion		614/2526 (24%)	–	
Tamponade		767/2974 (26%)	–	
Hypertension		9784/14641 (67%)	–	
Male		9622/16641 (58%)	–	

### Data extraction and analysis

All data was extracted pilot forms and entered into an excel database. For baseline variables, nominal data was recorded as the number of events (n) and expressed as a percentage. Continuous variables were expressed as mean and standard deviation, or median and interquartile ranges. Baseline patient data was aggregated. Medians and interquartile ranges were first converted to mean and standard deviation utilizing the method outlined by Hozo et al. [11]. Baseline patient data was compared between the octogenarian and non-octogenarian cohorts. A t-test was utilised in instances of continuous variables, and a two-sample test of proportions was utilised in dichotomous variables. Statistical significance was denoted as *P* < *0.05*. A meta-analysis of the primary and secondary outcomes was conducted and represented by forest plots where appropriate. A random effects model was used due to study heterogeneity and results expressed as either an odds ratio for binary data and *Hedges G* statistic for continuous data. Heterogeneity was assessed using the I^2^ test statistic. Low heterogeneity was denoted by I^2^ < 50%, moderate heterogeneity by I^2^ 50–74%, and high heterogeneity by I^2^ > 75%. Statistical analysis was carried out using Stata (Version 17.0, StataCorp, Texas, USA) ®

Kaplan–Meier survival curves were digitized where presented and an algorithmic computational tool was utilized to derive individual patient data as outlined by Guyot et al. [12]. Event and censoring data were compiled for 5 years, and overall survival curves were produced with Stata (Version 17.0, StataCorp, Texas, USA) ®. A log rank test was done to assess for significant survival differences between the groups, with significance denoted by *P* < 0.05. Studies that did not report numbers at risk for both groups were excluded from survival analysis as individual patient data (IPD) could not be derived.

### Assessment of bias

Some studies reported ‘consecutive recruitment’ of octogenarians, meaning that octogenarians received an operation regardless of age. Studies that did not report consecutive recruitment of octogenarians introduce a selection bias. The impact of this was assessed by performing a subgroup analysis of short-term mortality (outcome 1), comparing studies which reported consecutive recruitment to those which did not. A separate subgroup analysis of short-term mortality was also conducted stratifying studies published in Japan and elsewhere, in order to account for the impact of high baseline life expectancy encountered in the Japanese population. Publication bias was assessed through visual inspection of generated funnel plots and Begg’s rank correlation test R (R version 3.6.1, R Foundation for Statistical Computing, Vienna, Austria).

## Results

### Baseline characteristics

A total of 16 studies with 16,641 patients were included in the systematic review [13–28]. All papers were retrospective cohort studies. The number of included patients ranged from 101 to 5,175. Nine studies reported consecutive recruitment of octogenarians. The quality of included studies ranged from poor to good, with a total of six studies deemed as good quality. Baseline statistics, including reporting frequency are presented in Table 1. A total of 9,622 patients (58%) were male and 9784 (59%) had hypertension. Twenty-six percent and 24% of patients presented in tamponade or malperfusion respectively.

In terms of operative characteristics, cross clamp time (CCT) and cardiopulmonary bypass time (CPBT) was significantly lower in the octogenarian cohorts, at 110 and 167 min respectively (*P* = 0.04 and *P* = 0.02). Aortic root surgery and total arch replacements were formed significantly less frequently in the octogenarian cohorts, at 3.3% and 24% respectively (*P* < 0.01 and *P* = 0.01). Only one study commented on the use of frozen elephant trunk (FET) and this was not included in the analysis [17]. None of the studies commented on the rate of open distal anastomoses. Four studies commented on the deep hypothermic circulatory arrest times (DHCA) and use of antegrade cerebral perfusion (ACP). The mean DHCA time in the octogenarian cohort was 30.5 min (SD 12.9 min) however this was not significantly different from the non-octogenarian cohort (*P* = 0.37). The use of ACP in the octogenarian cohort was significantly lower (42% vs 59%, *P* = 0.04). Complete baseline data summarised in Additional file 1: table 2.

### Primary endpoint: morality

**Fig. 2:**
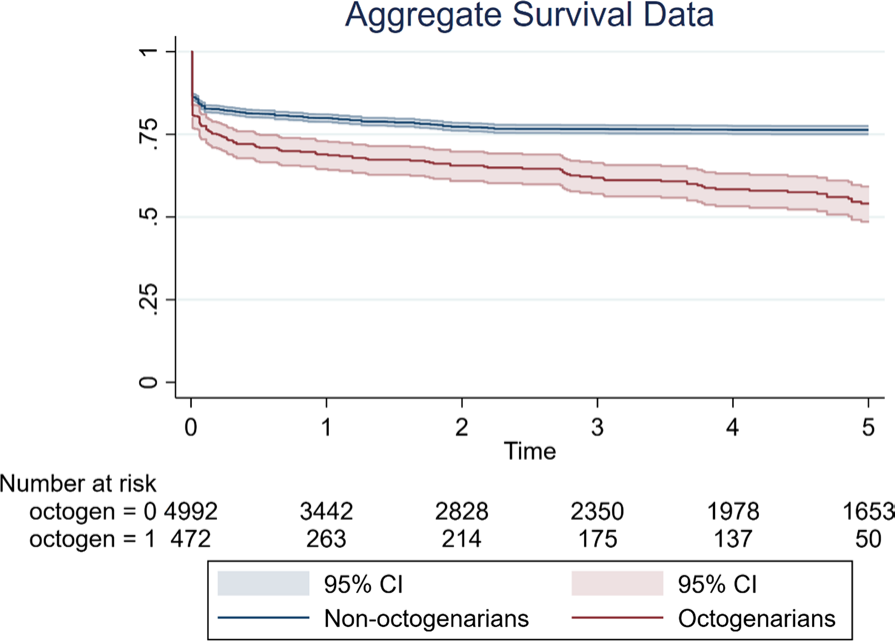
Aggregate mid-term survival (8 included studies)

**Fig. 3:**
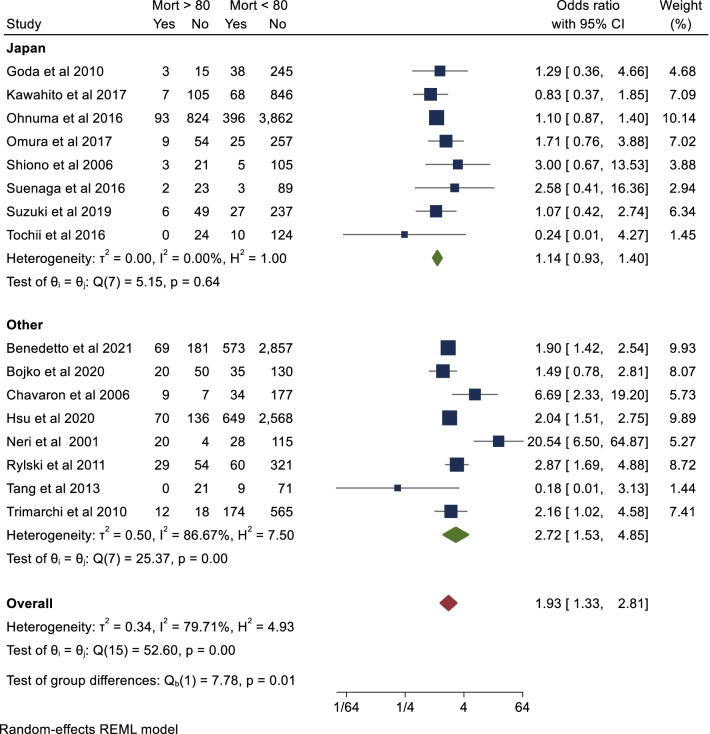
Short-term mortality based on country

**Fig. 4:**
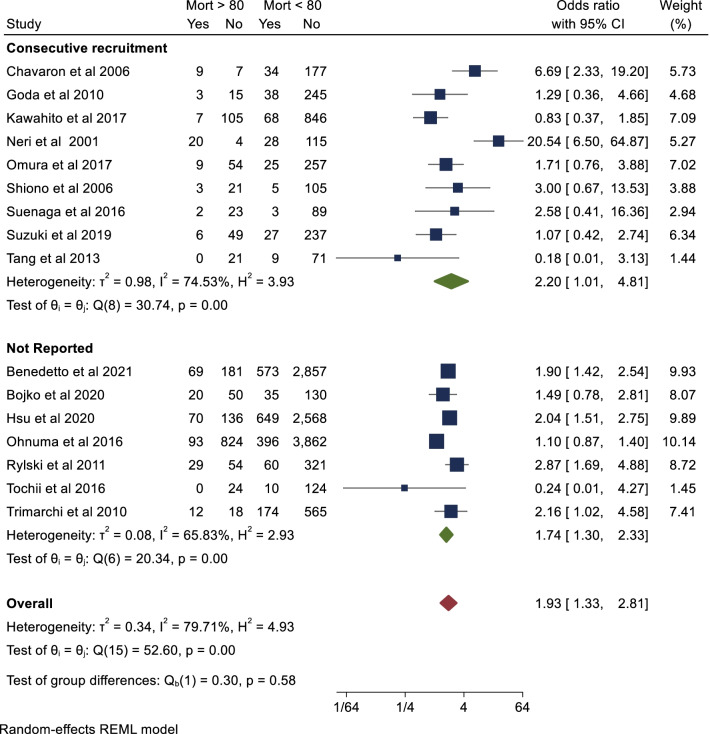
Short-term mortality based on study design (consecutive recruitment)

The primary endpoint of short-term mortality was reported in all included studies with a total of 16,641 patients. This is summarised in Additional file 1: table 3. Pooled analysis demonstrated that octogenarian cohorts are at significantly higher risk of short-term mortality than non-octogenarians (OR 1.93; 95% CI 1.33–2.81; *P* < 0.001). There was significant heterogeneity reported (I^2^ = 80%). There was no evidence of publication bias on visual inspection of funnel plots (Additional file 1: Fig. 2) or Begg’s test (*P* = 0.62). This result is represented in Fig. 1. Mid-term (five year) survival was reported in eight of the included studies. Seven studies provided Kaplan–Meier curves suitable for aggregation. Pooled analysis demonstrated that actuarial survival in yearly intervals to five years was 80%, 77%, 77%, 76% and 76% for the non-octogenarian group. Corresponding actuarial survival at the same intervals were 69%, 65%, 61% 58% and 54% in the octogenarian group respectively (Fig. 2). There was a significant difference in survival between both groups (*P* < 0.001). In terms of study location, there was a significantly lower odds ratio towards mortality in the octogenarian cohort in studies conducted in Japan to those conducted elsewhere (OR 1.14 vs 2.72, *P* = 0.01). Furthermore, short-term mortality was not significantly higher in the octogenarian cohort in the Japanese studies (*P* = 0.64) however was significantly higher in the non-Japanese studies (*P* < 0.01) (Fig. 3). In terms of study design, short term mortality was not significantly different when comparing studies that consecutively recruit octogenarians to those that do not (OR 2.20 vs 1.74, *P* = 0.58) (Fig. 4).

### Secondary endpoint: acute renal failure

At least one secondary endpoint was also reported in all included studies. These endpoints are summarised in Additional file 1: Table 3. Ten studies with a total of 11, 112 patients reported outcomes for post-operative ARF. The cumulative incidence in the octogenarian cohort was 11.7% compared to 12.8% on the non-octogenarian cohort. There was no significant difference between the two cohorts (OR 0.93; 95%CI 0.79–1.11; *P* = 0.44). There was no evidence of publication bias on visual inspection of funnel plot of odd ratio (Additional file 1: Fig. 3) (Begg’s test *P* = 0.62). Individually, none of the included studies demonstrate a significantly higher rate of postoperative renal failure in the octogenarian cohort.

### Secondary endpoint: postoperative stroke

Twelve studies with a total of 10,169 patients reported outcomes for post-operative stroke. The cumulative incidence of stroke in the octogenarian cohort was 9.4% compared to 10.5% in the non-octogenarian cohort. There was no significant difference between the cohorts (OR 0.92; 95%CI 0.71–1.21; *P* = 0.57). There was no evidence of publication bias on visual inspection of funnel plot (Additional file 1: Fig. 4) (Begg’s test *P* = 0.79).

### Secondary endpoint: re-exploration

Twelve studies with a total of 14,999 patients reported on re-exploration post-operatively. Octogenarians were at slightly higher risk of re-exploration post-operatively however this was not statistically significant (OR 1.08; 95%CI 0.81–1.45; *P* = 0.59). The breakdown for re-exploration was not specified in the included studies. There was asymmetry evident on visual inspection of forest plot (Additional file 1: Fig. 5a) confirmed on arcsine Begg test (*P* = 0.004). However, after correction for publication/small study bias with Duval and Tweedie trim and fill method there was still no significant difference in re-exploration post- operatively (corrected OR 1.12, 95% CI 0.75–1.67, *P* = 0.53 with additional imputed values (Additional file 1: Fig. 5b).

**Fig. 5:**
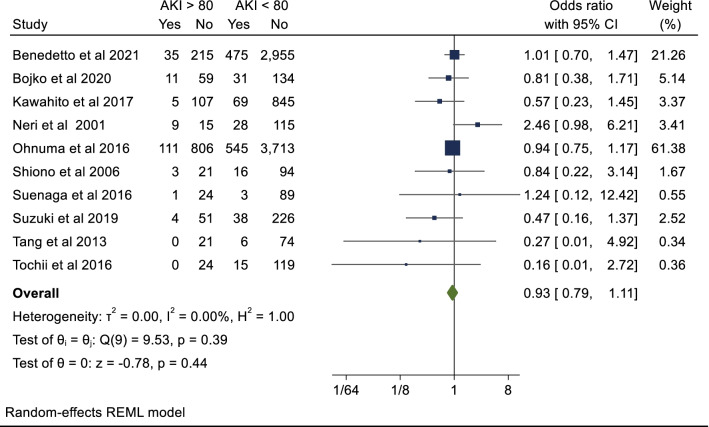
Postoperative acute renal failure

### Secondary endpoint: ICU length of stay

**Fig. 6:**
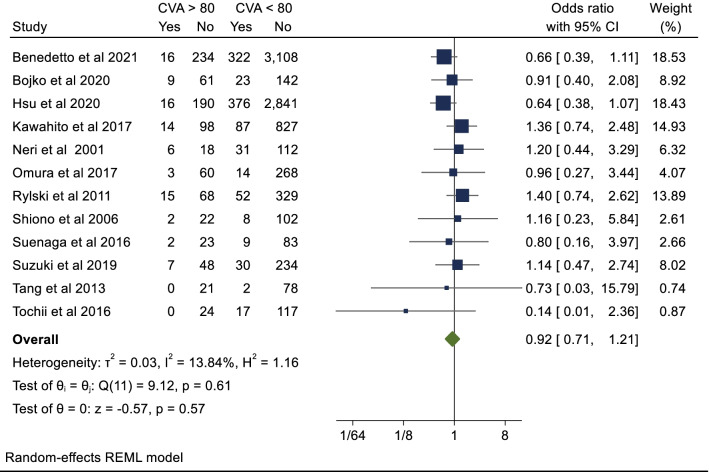
Postoperative stroke

**Fig. 7:**
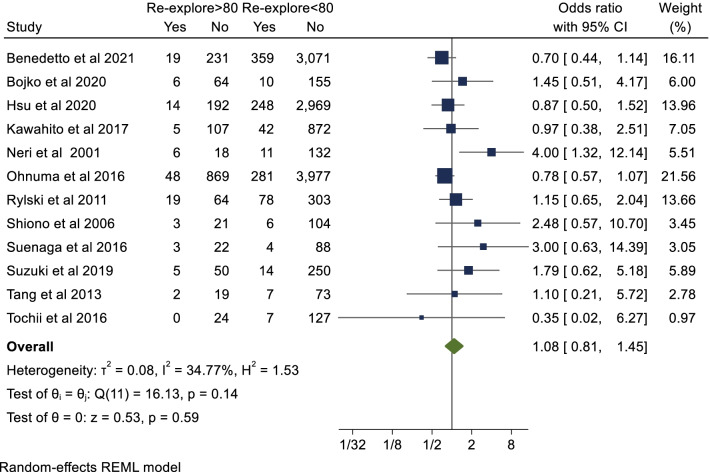
Re-exploration

**Fig. 8:**
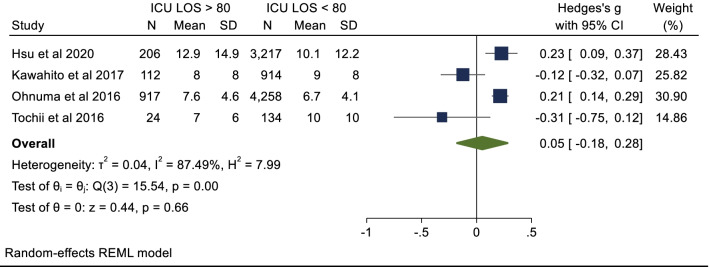
ICU length of stay

Four studies with a total of 9,782 patients reported data for ICU LOS (days). The mean ICU LOS in the octogenarian cohort was 7.7 days and 7.1 days in the non-octogenarian cohort. Hedges G statistic was 0.05 (95%CI − 0.18–0.28, *P* = 0.66) suggesting that there was no significant difference between the two cohorts. There was significant heterogeneity between the studies (I^2^ = 87%). These results are summarized in Figs. 5, 6, 7, 8.

## Discussion

The number of elderly patients undergoing surgery has risen in recent years due to the increasing age of the general population, technical improvements operatively and improvements in postoperative care [29]. In most forms of cardiac surgery, octogenarians demonstrate favorable long-term outcomes with low mortality and significantly improved post-operative quality of life [30, 31]. This is especially true in octogenarians undergoing aortic valve surgery [30]. ATAAD on the other hand, is associated with high mortality and morbidity and octogenarian status is a significant risk factor for mortality [4]. Concerns regarding post-operative quality of life and long-term outcomes means that this age demographic is more likely to be managed non-operatively [32, 33]. Aoyama et al. demonstrated that this transition point between patients managed operatively vs non-operatively is approximately 85 years [33]. Short-term mortality is significantly higher in non-operative management of octogenarians as patients pass away rapidly from the sequelae of malperfusion [28, 33]. Once the patient survives the initial event, survival in the long-term (10 years) of the operative cohort may be comparable to the non-operative cohort [34]. Patients and family members should be well informed of the risks of surgery and postoperative recovery.

The surgical approach between both cohorts differs, with a higher incidence of complex surgery in the younger group. Older patients were more likely to undergo isolated ascending aorta replacement rather than a composite graft implantation. Both groups underwent proximal or hemiarch replacement equally often, while younger patients underwent more total aortic arch replacements. Aortic root surgery was almost exclusively performed in the younger cohort, ranging from 1 to 5% in the octogenarian cohort across most studies. An aggressive surgical approach including a full root or arch replacement is associated with an improved freedom from reoperation rate and may not be associated with increased risk in an all-ages cohort, however, this may not be applicable to an elderly cohort [35, 36]. Elderly patients are particularly vulnerable to extended CPB and circulatory arrest times. In addition, the tissue quality may also be more friable making multiple anastomoses technically challenging. *Piccardo *et al. demonstrated that in an octogenarian cohort, the in-hospital mortality was prohibitively high in patients undergoing total arch replacement [37]. This approach may also seem unnecessary in an elderly cohort where long-term freedom from reoperation is not a pertinent issue [37]. Minimising CPB and circulatory arrest times in an octogenarian cohort is beneficial, by performing a less extensive procedure such as an ascending aorta replacement with an interposition graft [38]. Ghazy et al. termed this a ‘defensive strategy’ and demonstrated improved heath related quality of life (HR-QOL) when elderly patients are managed in this way [39]. Adopting a strategy aimed at minimising operative, CPB, circulatory arrest and rewarming time whilst ameliorating the life-threatening complications of ATAAD is recommended in the octogenarian cohort.

There was a significantly lower number of octogenarians patients undergoing antegrade cerebral perfusion (ACP) during ATAAD repair. Cohort studies demonstrate that ACP can be safely used in an elderly population [40–42]. *Pacini *et al. demonstrated a mortality of 7% and neurological event rate of 5% in 95 elderly patients (age > 75) undergoing aortic arch surgery, with ACP [40]. A limitation is that these outcomes may not extend to emergencies such as an ATAAD. Studies that assess ATAAD demonstrate a mortality benefit utilising the axillary artery as a preferred site of arterial inflow [42]. Future studies assessing an elderly population are warranted.

In the current study, early mortality was twice as high in the octogenarian cohort. This result is consistent with previous published systematic reviews. Biancari et al. (2011) demonstrated a pooled mortality of 36.7% in the octogenarian cohort, with octogenarians twice as likely to die in the short-term. Bruno et al. assessed an elderly population (age > 70), demonstrating an odds ratio of 2.25 and a pooled mortality rate of 19.9%. Since these two systematic reviews, a number of large contemporary retrospective studies have been published comparing octogenarians to non-octogenarians. This study demonstrated that octogenarians were twice as likely to die in the short-term after surgery for ATAAD. There are a number of possible factors that can explain this. Firstly, elderly patients tend to show fewer symptoms such as pain and present later, with more extensive dissections [20]. Secondly, perioperative complications are observed in higher frequency, such as cardiac tamponade, supra-aortic branch vessel involvement and dissections extending to the abdominal aorta [20, 22]. Octogenarians with risk factors such as malperfusion, heart failure or root involvement have a significantly higher mortality [17, 18, 43]. Thirdly, octogenarians do not have the same physiological reserve as younger patients, and do not recover from postoperative sequelae as easily. Our subgroup analysis of study location demonstrated a significantly lower mortality in Japanese studies compared to non-Japanese studies. Octogenarians account for 20% of patients undergoing surgery in Japan and this proportion is increasing [44]. The improved mortality may be a result of the high life expectancy of the Japanese population, lower incidence of atherosclerosis and extensive dissection [45]. Dissections involving this population are more likely to be less complex, Type-B with a retrograde component with fewer entry tears located in ascending aorta [45].

There were no significant differences in three out of the four of the secondary endpoints. The rate of stroke and ARF in both cohorts were comparable, with low heterogeneity between the studies. This result is consistent with previously published literature [8]. Though, the presence of postoperative ARF or stroke is associated with higher mortality in the elderly cohorts [46]. The results of ICU LOS were varied across the included studies and there was no significant difference between the two cohorts on pooled analysis. It should be noted that two high quality studies reported a longer ICU LOS in the elderly cohort [17, 20]. Elderly patients may require more time to wean from the ventilator and demonstrate protracted time to full neurological recovery postoperatively and therefore spend more time in ICU. None of the included studies demonstrated a significantly higher stroke rate in either population group. This result is in line with published data from large registry databases whereby the incidence of postoperative stroke was unrelated to age [47]. Causes of re-exploration include uncontrolled bleeding, tamponade and infection. This study did not demonstrate a significant difference in rates of re-exploration between either cohort. An element of selection bias may account for this, as there may have been higher threshold to manage unwell octogenarians (more prone to coagulopathy) operatively.

Actuarial survival was significantly lower in the octogenarian cohort, with a five-year survival in the octogenarian cohort of 54% compared to 76% in the non-octogenarian cohort. There was also significantly less attrition in the younger cohort over time. Studies report that the majority of deaths in the octogenarian cohort were caused by non-aortic events such as pneumonia and frailty, which may be more common postoperatively [18]. Two studies compared octogenarians post-surgical repair of ATAAD to an age-matched sample, demonstrating a significantly lower actuarial survival revealing the impact of surgery in this demographic [17, 26]. This current study demonstrated that octogenarians have a significantly lower mid-term survival after surgery for ATAAD, compared to non-octogenarians. Few studies compare non-operative to operative management of ATAAD in the elderly. In the short-term, survival of those surgically treated was superior [34, 43, 46]. There is a paucity of evidence assessing long-term outcomes of surgically treated ATAAD in the elderly cohort. The limited data available demonstrates that in the mid-term, outcomes are significantly worse in the non-operative arm, however, overall survival in the long-term (10 years) becomes comparable between both arms [34, 48, 49].

Two studies included in this review assessed HR-QOL outcomes in the elderly cohort [14, 26]. This is a useful indicator as it captures information on the physical and mental health status of a patient. Tang et al. compared octogenarians to non-octogenarians, demonstrating significantly lower physical functioning scores in the octogenarian cohort [26]. Bojko et al. demonstrated the same result, however this did not reach significance [14]. Studies that assess postoperative HR-QOL in the elderly following ATAAD repair also demonstrated significant attrition of physical health [50, 51]. Elderly patients have a greater degree of frailty and are also more vulnerable to the cerebral insult from deep hypothermic circulatory arrest which is required for some ATAAD repairs. As a result, the elderly cohort are more prone to lasting physical limitations of surgery compared to younger patients. There is a paucity of evidence assessing long term HR-QOL in octogenarians post-ATAAD repair. The availability of this data would be useful in making decision whether operative management is beneficial to octogenarians.

### Limitations

The main limitation of this study is the degree of study heterogeneity. All studies were retrospective in nature with inherent biases in design. A number of studies did not report whether octogenarians were recruited consecutively with resultant selection bias as only ‘well’ octogenarians may have been selected for surgery. Nine studies reported consecutive recruitment of octogenarians for surgery, with the exception of patients who were moribund or refused surgery [18]. This bias may have also extended to the recruitment of stable and robust octogenarians only. We aimed to reduce this bias by conducting a subgroup analysis of studies which stated consecutive recruitment of patients in the methodology. Furthermore, there was insufficient data reporting the use of open and closed distal anastomoses, DHCA times, FET in octogenarian populations, limiting analysis in this area. There was also variable reporting of cerebral perfusion strategies, which is an important measure as it has an impact on outcome [52]. Studies that ensure that relevant datapoints are collected pre- and post-operatively will minimize bias. Such studies are of course, difficult to facilitate in an emergent setting such as an ATAAD.

Population samples also demonstrate significant heterogeneity. All studies are from developed countries with significantly longer life expectancies, and therefore results of this study cannot be extrapolated to developing countries. The majority of studies come from Japan, which has a high life expectancy amongst developed countries [53]. Population samples also varied with time; six studies recruited patients from the 1990’s. Evidence suggests that operative mortality has improved significantly since these recruitment periods [6]. This is reflected in the mortality rate reported in these earlier studies, with studies recruiting from this period reporting a significantly higher mortality rate [15, 19]. We aimed to minimize this bias by including studies from 2000 onwards. Operative technique also varied significantly between the studies, where some studies adopted a defensive strategy and others had a high proportion of total arch repair and aortic root surgery in the elderly population. This would therefore have a considerable impact on primary and secondary endpoints.

## Conclusion

Octogenarians are twice as likely to die in the first 30-days following surgery for ATAAD and are almost twice as likely to require re-exploration postoperatively. The 5-year actuarial survival for octogenarians was 54% vs 76% for non-octogenarians. There were no significant differences in the incidence of postoperative stroke, renal failure and ICU LOS. Patients and family members should be well informed of the risks of surgery and postoperative recovery.

## Supplementary Information

**Additional file 1:** Supplementary data.

## Data Availability

The datasets used and/or analysed during the current study are available from the corresponding author on reasonable request.

## Abbreviations

ATAAD: Acute type A aortic dissection
ARF: Acute renal failure
ICU: Intensive care unit
LOS: Length of stay
PRISMA: Preferred Reporting Items for Systematic Reviews and Meta-Analyses
MeSH: Medical subject headings
IPD: Individual patient data
CCT: Cross clamp time
CPBT: Cardiopulmonary bypass time
DHCA: Deep hypothermic circulatory arrest
FET: Frozen elephant trunk
ACP: Antegrade cerebral perfusion
HR-QOL: Heath related quality of life
