# Supplementary material for: Outcomes of surgery for acute type A dissection in octogenarians versus non-octogenarians: a systematic review and meta analysis
Source: J Cardiothorac Surg. 2022 Sep 1;17:222. doi: 10.1186/s13019-022-01980-x (PMC9434858; doi:10.1186/s13019-022-01980-x)
Supplement: Supplementary file 1 — Additional file 1: Supplementary data. [file 13019_2022_1980_MOESM1_ESM.docx]

Records excluded

N = 474

Full-text articles assessed for eligibility
n = 75

Records screened
n = 549

Records after duplicates removed
n = 549

Additional records identified through reference check

N = 4

Records Identified through Database Searches
n = 596

## Identification

## Screening

## Eligibility

Reasons for exclusion (59)

Full text not available (2)

Wrong study design (39)

Wrong comparison (3)

Wrong age thresholds (13)

Wrong indication (2)

Studies included in qualitative synthesis

N = 16

## Included

**Supplementary Figure 1.** PRISMA flow-chart summarizing the search strategy for relevant publications.

| *Supplementary table 1: Study Quality* | | | | | | | | | | |
| --- | --- | --- | --- | --- | --- | --- | --- | --- | --- | --- |
| *Authors* | Years of Inclusion | Country | Study Design | Was the primary outcome a comparison | Octogenarian Baseline Characteristics Reported | Operative details provided | Cerebral protection method provided | Consecutive Recruitment of Octogenarians | Long term follow up reported | Overall Quality |
| Benedetto et al 2021 | 2009-2018 | UK | R | No | No | No | No | NR | No | Poor |
| Bojko et al 2020 | 2002-2017 | USA | R | Yes (octogenarians to septuagenarians) | Yes | Yes | No | NR | Yes | Average |
| Chavaron et al 2006 | 1990-2005 | France | R | Yes | No | Yes | No | Yes | No | Average |
| Goda et al 2010 | 1997-2007 | Japan | R | No | No | Yes (not age stratified) | Yes (not age stratified) | Yes | No | Poor |
| Hsu et al 2020 | 2005-2013 | Taiwan | R | Yes | Yes | Yes | No | NR | Yes | Good |
| Kawahito et al 2017 | 1990-2016 | Japan | R | Yes | Yes | Yes | No | Yes | Yes | Good |
| Neri et al 2001 | 1985-1999 | France | R | Yes | Yes | Yes | Yes | Yes | No | Average |
| Ohnuma et al 2016 | 2011-2013 | Japan | R | Yes | Yes | Yes | No | NR | No | Average |
| Omura et al 2017 | 2001-2015 | Japan | R | Yes | Yes | Yes | Yes | Yes | Yes | Good |
| Rylski et al 2011 | 2006-2009 | Germany | R | Yes | Yes | Yes | No | NR | No | Average |
| Shiono et al 2006 | 1995-2005 | Japan | R | Yes | Yes | Yes | No | Yes | Yes | Good |
| Suenaga et al 2016 | 2000-2013 | Japan | R | Yes | Yes-minimal | No | No | Yes | Yes | Average |
| Suzuki et al 2019 | 2004-2017 | Japan | R | Yes | Yes | Yes | No | Yes | Yes | Good |
| Tang et al 2013 | 2005-2011 | USA | R | Yes | Yes | Yes | No | Yes | Yes | Good |
| Tochii et al 2016 | 2005-2015 | Japan | R | Yes | Yes-minimal | Yes | No | NR | Yes | Average |
| Trimarchi et al 2010 | 1996-2004 | Multicentre | R | Yes (primarily over 70 to under 70) | No | No | No | NR | No | Poor |

| Supplementary Table 2: Preoperative and Operative Patient Characteristics | | | | | | | | | | | | | | | | | | |
| --- | --- | --- | --- | --- | --- | --- | --- | --- | --- | --- | --- | --- | --- | --- | --- | --- | --- | --- |
| *Authors* | CCT > 80  (min) | CCT < 80  (min) | CPBT > 80  (min) | CPBT < 80  (min) | Root replacement > 80 | Root replacement < 80 | Arch Replacement > 80 | Arch Replacement < 80 | FET > 80 | FET < 80 | DHCA > 80 | DHCA < 80 | ACP > 80 | ACP < 80 | Malperfusion | Tamponade | HTN | Male |
| Benedetto et al 2021 | - | - | - | - | - | - | - | - | - | - | - | - | - | - | - | - | 2483 (67%) | 2464 (70%) |
| Bojko et al 2020 | 141 (113-159) | 143 (113-175) | 197 (172-230) | 211 (180-250) | 6 (3%) | 33 (14%) | 1 (<1%) | 14 (6%) | - | - | 31 (27-39) | 35 (27-39) | - | - | 53 (23%) | 36 (15.3%) | 213 (91%) | 112 (48%) |
| Chavaron et al 2006 | **-** | **-** | **-** | **-** | - | - | - | - | - | - | - | - | - | - | 19 (8.8%) | 37 (17%) | 121 (56%) | 150 (69%) |
| Goda et al 2010 | **-** | **-** | **-** | **-** | - | - | - | - |  |  |  |  |  |  | - | 83 (28%) | 196 (65%) | 163 (54%) |
| Hsu et al 2020 | - | - | - | - | 13 (6%) | 347 (11%) | 67 (33%) | 945 (29%) | 1 (0.5%) | 85 (2.6%) | - | - | - | - | - | - | 2355 (69%) | 2351 (69%) |
| Kawahito et al 2017 | - | - | - | - | 1 (1%) | 53 (6%) | 12 (11%) | 161 (18%) |  |  |  |  |  |  | 313 (31%) | 236 (23%) | - | 534 (52%) |
| Neri et al 2001 | 71 ± 27 | 65 ± 33 | 178 ±28 | 167 ±31 | 6 (25%) | 37 (21%) | 3 (12%) | 37 (22%) | - | - | 32 ±11 | 31 ±12 | 0 | 29 (17%) | - | 91 (46%) | 178 (90%) | 130 (66%) |
| Ohnuma et al 2016 | - | - | - | - | 16 (1.7%) | 132 (3.1%) | 275 (30%) | 1874 (44%) | - | - | - | - | - | - | - | - | 3157 (61%) | 2639 (51%) |
| Omura et al 2017 | 101 ± 32 | 124 ± 51 | 168 ± 48 | 192 ± 72 | 0 | 19 (6.7%) | 9 (14.3%) | 86 (30.4%) | - | - | - | - | - | - | 79 (23%) | - | - | 178 (52%) |
| Rylski et al 2011 | - | - | - | - | 9 (11%) | 56 (15%) | 8 (9%) | 54 (14%) | - | - | 29 ± 18.7 | 29.7 ± 20.3 | 55 (66.27%) | 289 (75.85%) | - | 115 (25%) | - | 210 (45%) |
| Shiono et al 2006 | 112 ± 34 | 137 ± 51 | 185 ± 35 | 218 ± 60 | 0 | 9 (8.2%) | 1 (4.2%) | 28 (25%) | - | - | - | - | 24 (100%) | 110 (100%) | - | 57 (43%) | 119 (89%) | 62 (46%) |
| Suenaga et al 2016 | 70 ± 15 | 70 ± 17 | 150 ± 32 | 144 ± 19 | - | - | - | - |  |  | 30 ±4.9 | 33 ± 7.6 | - | - | 22 (28%) | - | 54 (47%) | 24 (30%) |
| Suzuki et al 2019 | - | - | 111 (101 – 126) | 109 (93 – 137) | 0 | 7 (2.7%) | 4 (7.3%) | 19 (7.2%) | - | - | - | - | 0 | 0 | 82 (26%) | 91 (29%) | 200 (63%) | 155 (49%) |
| Tang et al 2013 | 94 ± 42 | 103 ± 49 | 177 ± 51 | 186 ± 48 | 1 (5%) | 23 (29%) | 0 | 2 (3%) | - | - | - | - | - | - | 33 (33%) | 21 (21%) | 79 (78%) | 63 (62%) |
| Tochii et al 2016 | 135 ± 27 | 161 ± 53 | 193 ± 31 | 235 ± 68 | 0 | 18 (13%) | 1 (4%) | 36 (27%) | - | - | - | - | - | - | 13 (8%) | - | - | 75 (49%) |
| Trimarchi et al 2010 | - | - | - | - | - | - | - | - | - | - | - | - | - | - | - | - | 629 (67%) | 312 (33%) |

| Supplementary table 3: Primary and Secondary Endpoint data | | | | | | | | | | | | |
| --- | --- | --- | --- | --- | --- | --- | --- | --- | --- | --- | --- | --- |
| *Authors* | # > 80 surgically managed | # < 80 Surgically managed | Early Mortality > 80 | Early Mortality < 80 | CVA > 80 | CVA < 80 | AKI > 80 | AKI < 80 | Re-exploration > 80 | Re-exploration < 80 | ICU LOS > 80 | ICU LOS < 80 |
| Benedetto et al 2021 | 250 (7%) | 3430 (93%) | 69 (27.6%) | 573 (17%) | 16 (6.4%) | 322 (9%) | 35 (15.2%) | 475 (14%) | 19 (8.7%) | 359 (10%) | NR | NR |
| Bojko et al 2020 | 70 (30%) | 165 (70%) | 20 (28.6%) | 35 (21.2%) | 9 (12.9%) | 23 (13.9) | 11 (15.7%) | 31 (18.8%) | 6 (8.6%) | 10 (6.1%) | NR | NR |
| Chavaron et al 2007 | 16 (56%) | 211 (16%) | 9 (56%) | 34 (16%) | NR | NR | NR | NR | NR | NR | NR | NR |
| Goda et al 2010 | 18 (6%) | 283 (94%) | 3 (17%) | 38 (13%) | NR | NR | NR | NR | NR | NR | NR | NR |
| Hsu et al 2020 | 206 (6%) | 3217 (94%) | 70 (34%) | 649 (20.2%) | 16 (7.8%) | 376 (11.7%) | NR | NR | 14 (6.8%) | 248 (7.7%) | 12.9+-14.9 | 10.1 +-12.2 |
| Kawahito et al 2017 | 112 (11%) | 914 (89%) | 7 (6.3%) | 68 (7.4%) | 14 (12.5%) | 87 (9.5%) | 5 (4.5%) | 69 (7.5%) | 5 (4.5%) | 42 (4.6%) | 8 +- 8 | 9 +- 8 |
| Neri et al 2001 | 24 (12%) | 143 (88%) | 20 (83%) | 28 (16%) | 6 (25%) | 31 (18%) | 9 (37%) | 28 (16%) | 6 (25%) | 11 (6%) | NR | NR |
| Ohnuma et al 2016 | 917 (18%) | 4258 (82%) | 93 (10.1%) | 396 (9.3%) | NR | NR | 111 (12.1%) | 545 (12.8%) | 48 (5.2%) | 281 (6.6%) | 7.6 +-4.6 | 6.7 +- 4.1 |
| Omura et al 2017 | 63 (18%) | 282 (82%) | 9 (14.3%) | 25 (8.9%) | 3 (4.8%) | 14 (5%) | NR | NR | NR | NR | NR | NR |
| Rylski et al 2011 | 83 (35%) | 381 (16%) | 29 (34.9%) | 60 (15.7%) | 15 (18.1%) | 52 (13.6%) | NR | NR | 19 (22.9%) | 78 (20.5%) | 12.5 (161) | 10.2 (NR) |
| Shiono et al 2006 | 24 (18%) | 110 (82%) | 3 (12.5%) | 5 (5.5%) | 2 (8.3%) | 8 (7.3%) | 3 (12.5%) | 16 (14.5%) | 3 (12.5%) | 6 (5.5%) | NR | NR |
| Suenaga et al 2016 | 25 (21%) | 92 (79%) | 2 (8%) | 3 (5%) | 2 (8%) | 9 (16%) | 1 (4%) | 3 (5%) | 3 (12%) | 4 (7%) | NR | NR |
| Suzuki et al 2019 | 55 (17%) | 264 (83%) | 6 (10.9%) | 27 (10.2%) | 7 (12.7%) | 30 (11.4%) | 4 (7.3%) | 38 (14.4%) | 5 (9.1%) | 14 (5.3%) | NR | NR |
| Tang et al 2013 | 21 (21%) | 80 (79%) | 0 | 9 (11.2%) | 0 | 2 (3%) | 0 | 6 (8%) | 2 (10%) | 7 (9%) | NR | NR |
| Tochii et al 2016 | 24 (15%) | 134 (85%) | 0 | 10 (7.5%) | 0 | 17 (12.7%) | 0 | 15 (11.2%) | 0 | 7 (5.2%) | 7 +-6 | 10 +- 10 |
| Trimarchi et al 2010 | 30 (4%) | 739 (96%) | 12 (6.4%) | 174 (23.5%) | NR | NR | NR | NR | NR | NR | NR | NR |


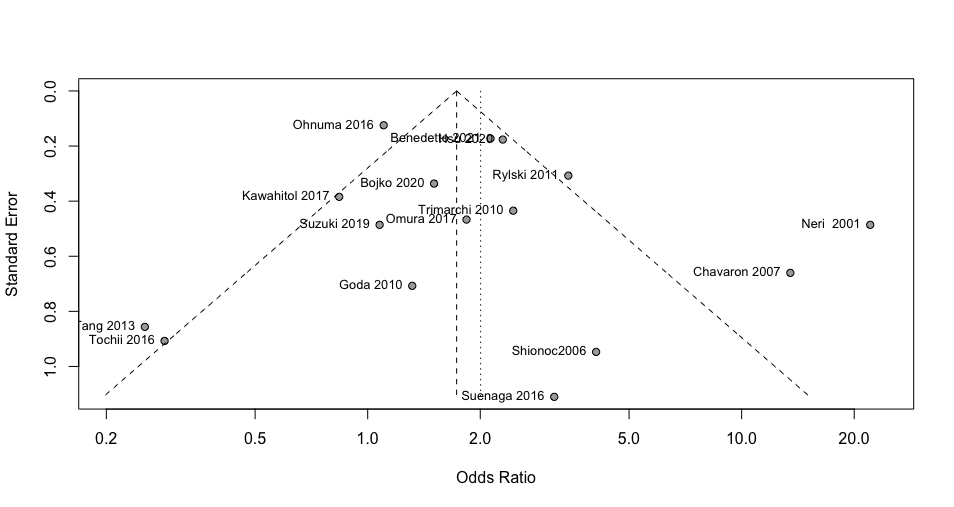


Supplementary figure 2. Funnel plot of impact of age on early mortality post-acute type A aortic dissection repair


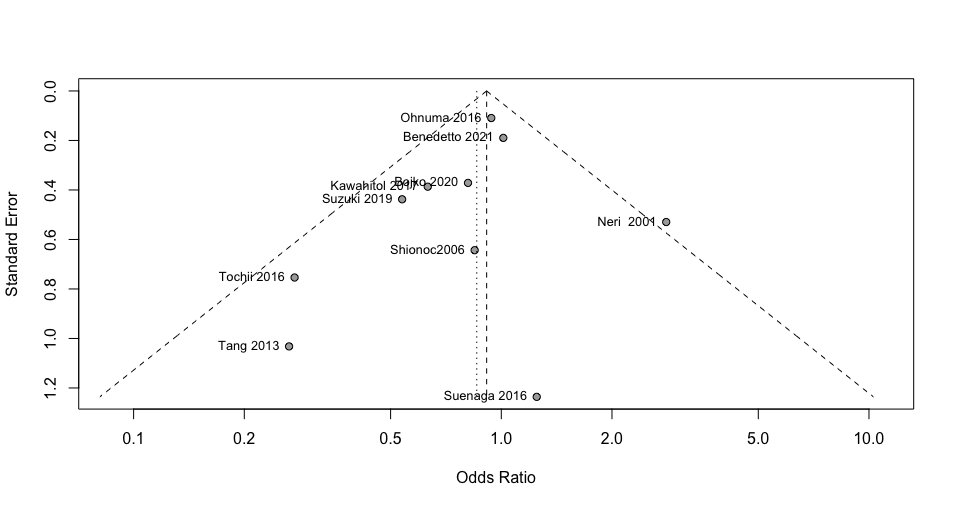


Supplemental figure 3. Funnel plot of impact of age on occurrence of acute renal failure post-acute type A aortic dissection repair


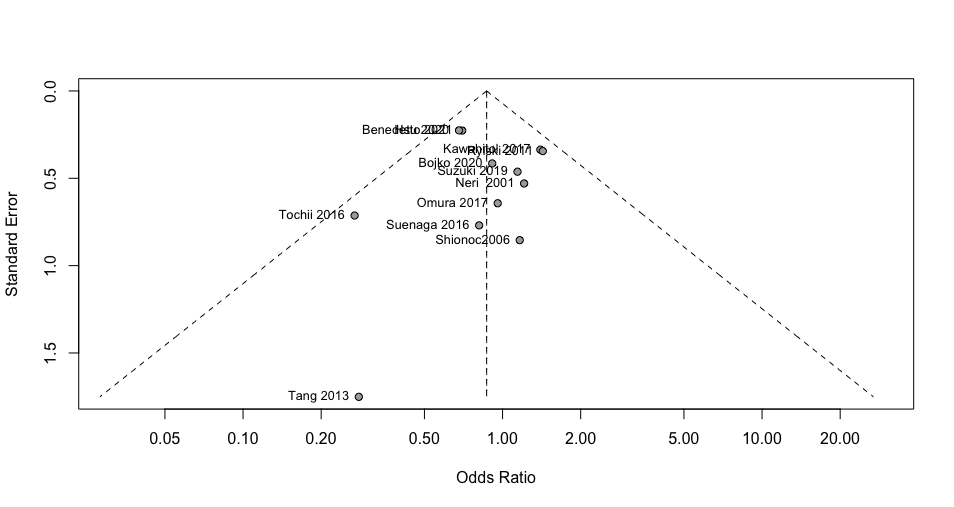


Supplemental figure 4. Funnel plot of impact of age on occurrence of stroke post-acute type A aortic dissection repair


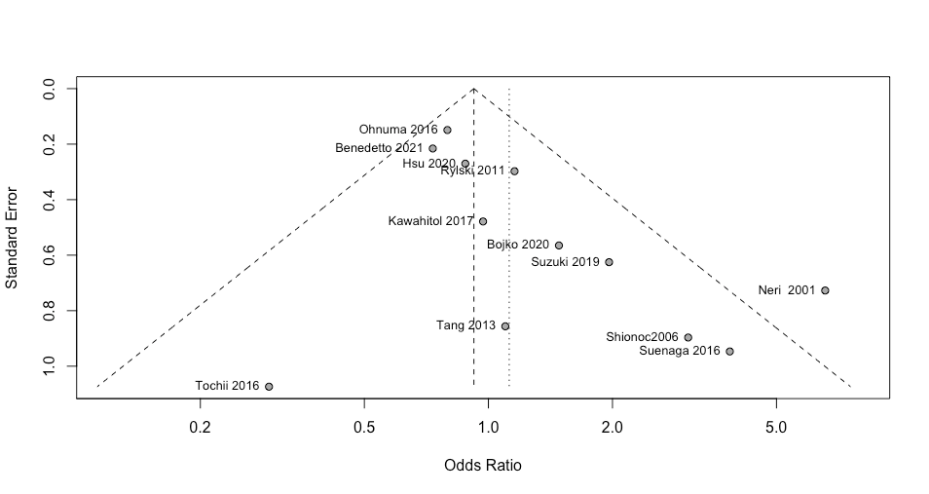

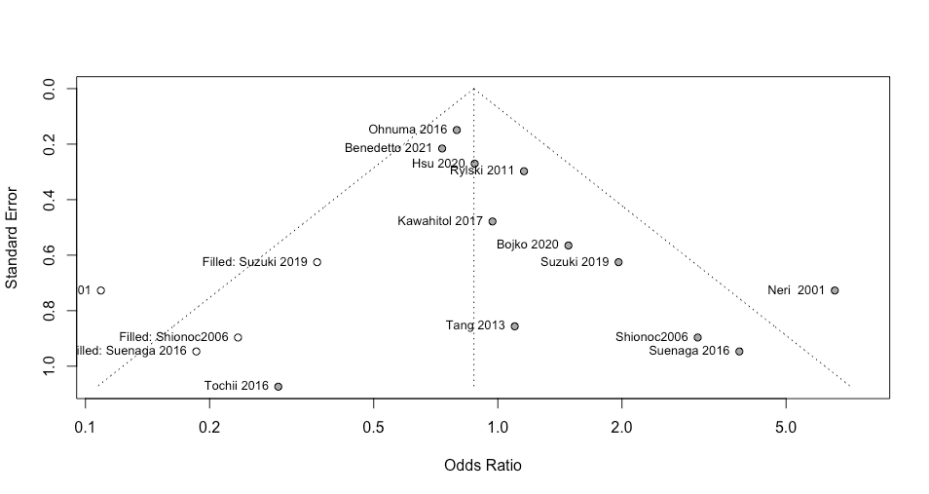


b

a

Supplemental figure 5. A – Unconnected Funnel plot of impact of age on occurrence of re-exploration post-acute type A aortic dissection repair, B – Trim and fill funnel plot of impact of age on occurrence of re-exploration post-acute type A aortic dissection repair
